# Topological Atomic Chains on 2D Hybrid Structure

**DOI:** 10.3390/ma14123289

**Published:** 2021-06-14

**Authors:** Tomasz Kwapiński, Marcin Kurzyna

**Affiliations:** Department of Physics, Maria Curie-Skłodowska University, PL-20031 Lublin, Poland; marcin.kurzyna@live.umcs.edu.pl

**Keywords:** atomic chain, Su-Schrieffer-Heeger model, density of states, van Hove singularities, localized states, hybrid systems

## Abstract

Mid-gap 1D topological states and their electronic properties on different 2D hybrid structures are investigated using the tight binding Hamiltonian and the Green’s function technique. There are considered straight armchair-edge and zig-zag Su–Schrieffer–Heeger (SSH) chains coupled with real 2D electrodes which density of states (DOS) are characterized by the van Hove singularities. In this work, it is shown that such 2D substrates substantially influence topological states end evoke strong asymmetry in their on-site energetic structures, as well as essential modifications of the spectral density function (local DOS) along the chain. In the presence of the surface singularities the SSH topological state is split, or it is strongly localized and becomes dispersionless (tends to the atomic limit). Additionally, in the vicinity of the surface DOS edges this state is asymmetrical and consists of a wide bulk part together with a sharp localized peak in its local DOS structure. Different zig-zag and armachair-edge configurations of the chain show the spatial asymmetry in the chain local DOS; thus, topological edge states at both chain ends can appear for different energies. These new effects cannot be observed for ideal wide band limit electrodes but they concern 1D topological states coupled with real 2D hybrid structures.

## 1. Introduction

Nowadays, one can notice a growing interest of scientists in low-dimensional structures which find almost constantly new applications in many branches of science and technology, e.g., in nanoelectronics [1,2], biotechnology, engineering [3], chemistry [4] and medicine [5]. Atomic wires as the thinnest possible electrical conductors are especially interesting objects to study mainly from practical point of view. Their electronic properties are the subject of many theoretical and experimental papers as they reveal a great deal of interesting physical phenomena which are often hard to notice in bulk materials, e.g., spin-charge separation [6,7], Majorana topological states [8,9], charge-density waves [10,11,12], turnstile effects, photon-assisted tunneling and pumping effects, or coherent destruction of tunneling [13,14,15,16,17,18,19,20]. In low-dimensional structures one can also observe unique solid-state phases such as time crystals [21,22,23], transient crystals [24] or Floquet topological  insulators [25,26]. Especially interesting are hybrid systems composed of different low-dimensional structures where quantum states of one structure could penetrate to the second one leading to a kind of proximity effect which was studied, e.g., in one-dimensional (1D) coupled systems [27,28].

The main topic of interest in this work are the electrical properties of 1D topological chains which are fabricated on a flat two-dimensional (2D) electrode or which are placed between two 2D leads. Such a hybrid system can reveal constructive or destructive coexistence of 1D and 2D features of different quantum structures thus it is desirable to investigate their properties thoroughly. Topological chains are special materials where energy gaps appear inside the system and the edge states (topological states) are observed at the system boundaries. Such materials have unique electrical properties, i.e., topological states are insensitive to external perturbations, they survive for different substrates, reveal long-range conductance oscillations and can play a role of effective electron pumps [11,20]. Simple topological phases in one-dimensional chains can be obtained within a fermionic Su–Schrieffer–Heeger (SSH) model [29,30]. The model possesses two different topological phases: the trivial phase with an energy gap along the whole system, and the nontrivial one with spectrally isolated mid-gap states at the system boundaries.

In this paper we analyse modified SSH chains (straight, zig-zag and armchair edge geometries) in contact with different types of 2D electrodes which play a role of electron reservoirs. The leads can be characterized by a flat band structure or can possess van Hove singularities in their density of states (DOS). Such singularities (peaks or dips in DOS) are commonly found in real 2D structures like graphene, silicene, antimonene or other atomic layers that could be fabricated and modified to obtain fully functional materials [2,31,32]. Although topological insulators, as well as topological 1D structures have been intensively investigated in last years the influence of low-dimensional electrodes on topological states in hybrid systems has been often overlooked. The main objective of this work is to find how topological SSH states are influenced by the DOS singularities of 2D substrates. It is crucial to verify if these states are robust to these singularities and if the space and energetic symmetries of 1D edge states (which are responsible for the particle-hole symmetry) survive in the presence of 2D substrates. Moreover, the question if the SSH topological states can appear outside the electrode’s DOS (beyond the band) is going to be answered in this paper.

## 2. Model and Theoretical Description

**Figure 1:**
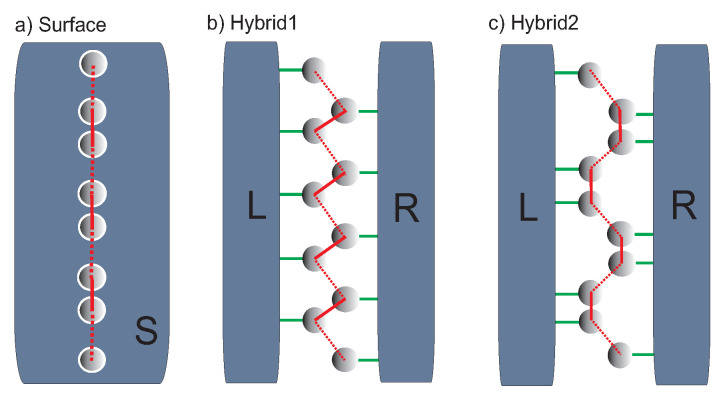
Schematic view of the SSH atomic chain for different geometries: (**a**) straight chain on 2D electrode, (**b**,**c**) zig-zag and armchair edge chains between two 2D electrodes called here Hybrid1 and Hybrid2, respectively. The broken and solid red lines represent different couplings between the nearest-neighbour atomic sites in the SSH chain.

Hybrid systems under consideration are shown in Figure 1. In the simplest case there is a straight SSH chain on 2D electrode (panel a). The surface is described by the tight-binding Hamiltonian for a regular atomic lattice (e.g., rectangular or hexagonal) which leads to the effective surface DOS with the van Hove singularities (see Appendix A). The hybrid systems (Hybrid1 and Hybrid2) are depicted in panels (b) and (c) in Figure 1, where a zig-zag (or armchair edge) SSH chain is placed between two 2D electrodes. In this case, the electronic structure of the chain is influenced by two different electron reservoirs (hybrid structures) which is the central point of studies prested in this paper.

Such systems can be realized experimentally using modified vicinal surfaces where a single or double chains as well as atomic ribbons can be easily fabricated within the epitaxy method. These structures can be investigated by means of the STM (Scanning Tunneling Microscope) technique which measures current-voltage characteristics or the conductance that is proportional to the local DOS. Detailed analysis of this quantity allows one to distinguish different topological phases of the SSH chains [33,34]. Among many experimental fabrication methods of 1D systems one can find atomic chains grown epitaxially on silicon surfaces, such as Si(335), Si(557) [35,36,37], 1D chains with gate-defined quantum dot in 2D electron gas or chains of dopant atoms in silicon [38,39]. Alternatively, the SSH topology was found in chlorine vacancies on Cu(100) surfaces [34,40]. In such systems, the role of 2D substrate electrodes can play the surface atomic reconstruction (like Si(111)-(6 × 6)Au surface) or atomically flat graphene, silicene, antimonene or other layers which nowadays can be easily fabricated [2,31,32,41,42]. It is also possible to combine one-dimensional SSH chains between two 2D substrates (hybrid geometry). In such a case, the chain localized at the step of the surface is coupled with both nearest terraces (they stand for 2D structures with different DOS). Alternatively, one can use precisely controlled quantum dot chain coupled with different electrodes characterized by the van Hove peaks.

The tight-binding model Hamiltonian in the standard second-quantized notation for the system shown in Figure 1, can be written as follows: $H=H_{0}+H_{coup}$, where:

(1) $$\begin{array}{ccc} H_{0} & = & \sum_{i=1}^{N}\varepsilon_{i}a_{i}^{†}a_{i}+\sum_{\alpha }\sum_{\vec{k}}\varepsilon_{\vec{k}\alpha }a_{\vec{k}\alpha }^{†}a_{\vec{k}\alpha } \end{array}$$

describes the on-site electron energies in the atomic chain, $\varepsilon_{i}$, and in the α electrode, $\varepsilon_{\vec{k}\alpha }$, with the wave vectors $\vec{k}$ (α=S corresponds to only one surface electrode (Figure 1, panel a), and α=L,R corresponds to the hybrid geometries shown in Figure 1, panels b and c). The operators $a_{i}\left(a_{i}^{†}\right)$ annihilate(create) an electron at *i*-th site of the chain (i=1,...,N) and $a_{\vec{k}\alpha }\left(a_{\vec{k}\alpha }^{†}\right)$ are the according leads annihilation(creation) operators. In calculations both spin directions are independent of each other and the spin index in operators is suppressed. As we are interested in the SSH topological state modifications, in the first step it is assumed that the electron correlation effects do not play an important role. This assumption is reasonably well satisfied, e.g., for lead or gold chains on vicinal silicon surfaces. For small electron-electron correlation it can be captured by an effective shift of the chain onsite energies [43,44,45]. The electron transitions along the chain and between the surface and chain sites are described by the coupling Hamiltonian in the following form:

(2) $$\begin{array}{ccc} H_{coup} & = & \sum_{i=1}^{N-1}V_{i,i+1}a_{i}^{†}a_{i+1}+\sum_{i=1}^{N}\sum_{\vec{k}\alpha }V_{i,\vec{k}\alpha }a_{\vec{k}\alpha }^{†}a_{i}+H.c. \end{array}$$

The first part of this Hamiltonian consists of the tunneling matrix elements $V_{i,i+1}$ which describe the couplings between the neighboring sites in the chain. Note that for different every second couplings $V_{i,i+1}$ ($V_{i,i+1}=V,V_{i+1,i+2}=W$ such that V≠W) the system corresponds to the topological SSH chain [46,47,48] and for equally coupled sites (V=W) the system is in the normal state. By taking V<W one gets a topological chain in the nontrivial phase (called here SSH_1_), i.e., with topological mid-gap states at both chain ends. When one applies V>W it is obtained a chain in the trivial topological phase without end states (SSH_0_). The second part of Equation (2) describes the chain-substrate coupling where electron transitions between the *i*-th chain state and the surface states are established by $V_{i,\vec{k}\alpha }$ matrix elements (hybridization terms).

In this paper the topic of interest is the spectral density function at atomic sites in the chain (local DOS) which can be obtained from the knowledge of the corresponding diagonal matrix elements of the retarded Green’s operator at a given site [49,50]:

(3) LDOSi(E)=−1πIm(Giir(E+)),

where $E^{+}=E+i\varepsilon$ (ε is positive infinitesimal number which tends to zero) and $G_{ii}^{r}\left(E\right)$ for the time-independent Hamiltonian satisfies the following equation of motion:

(4) $$\begin{array}{c} EG_{ij}^{r}\left(E\right)=\langle {\left[a_{i},a_{j}^{†}\right]}_{+}\rangle +{\langle \langle {\left[a_{i},H\right]}_{-};a_{j}^{†}\rangle \rangle }_{E}, \end{array}$$

where the bracket ${\langle \langle ...\rangle \rangle }_{E}$ corresponds to new energy-dependent Green’s function which includes $\vec{k}\alpha$ states due to the total Hamiltonian in the above equation. For *N*-site system the total Green’s function could be obtained by solving *N* coupled linear equations which can be written in the matrix notation: ${\hat{G}}^{r}\cdot \hat{A}=I$, where I is the identity matrix. The matrix ${\hat{G}}^{r}$ stands for N×N array of the appropriate Green’s functions and $\hat{A}$ is a square N×N complex array which can be found from Equation (4) and it takes the following form:

(5) $$\begin{array}{ccc} A_{i,j}^{N\times N}\left(E\right) & = & \left(E-\varepsilon_{i}\right)\delta_{i,j}-V_{i,j+1}\left(\delta_{i,j+1}+\delta_{i+1,j}\right)+\Sigma_{i,j}\left(E\right), \end{array}$$

where $\Sigma_{i,j}\left(E\right)=\sum_{\vec{k}\alpha }\frac{V_{i,\vec{k}\alpha }^{*}V_{j,\vec{k}\alpha }}{E^{+}-\varepsilon_{\vec{k}\alpha }}$. The off-diagonal elements of the matrix $\hat{\Sigma }\left(E\right)$ depend exponentially on *i*-th and *j*-th atomic distance [45,51] so they rapidly vanish and in the paper are negligible. It allows one to consider the electrode as a set of equivalent leads such that each chain site is coupled with its own electrode. Thus, assuming that only diagonal terms of the matrix $\hat{\Sigma }\left(E\right)$ play an important role (and they are the same for all chain sites, $\Sigma_{i,j}\left(E\right)=\delta_{ij}\Sigma \left(E\right)$) one can express this function by the real and imaginary complex forms as Σ(E)=Λ(E)−iΓ(E)/2. Here $\Gamma \left(E\right)=2\pi |V_{\vec{k}\alpha }{|}^{2}\sum_{\vec{k}\alpha }\delta \left(E-\varepsilon_{\vec{k}\alpha }\right)=2\pi {\left|V_{\vec{k}\alpha }\right|}^{2}DOS_{\alpha }\left(E\right)$, where $DOS_{\alpha }\left(E\right)$ stands for energy dependent α lead (surface) DOS. Both functions Λ(E) and Γ(E) are not independent, see Appendix B, and they are responsible for the localized states in the system which appear for some values of the chain on-site energies. For a flat and $\vec{k}$-independent surface DOS, i.e., within the wide band approximation one has: Λ(E)=0 and Γ(E)=Γ which is constant and energy independent [44,45,49,51]. On the other hand, assuming a specific surface DOS like for 2D materials (see Appendix A for the 2D van Hove DOS) one can find Γ(E) function and then using the Hilbert transform can obtain the second Λ(E) function. It allows one to write explicitly all matrix elements of $\hat{A}$, Equation (5), and the retarded Green’s functions can be obtained by inversion of this matrix, i.e.,

(6) $$\begin{array}{c} G_{ii}^{r}\left(\varepsilon \right)={\left({\hat{A}}^{-1}\right)}_{ii}=\frac{\mathrm{cof}{\hat{A}}_{ii}}{\det\hat{A}}\;, \end{array}$$

where $\mathrm{cof}\hat{A}$ and $\det\hat{A}$ mean the cofactor (algebraic complement) and determinant of the matrix $\hat{A}$. For a regular chain with the same atom-atom couplings and homogenous on-site energies these functions can be expressed analytically by means of the Chebyshev polynomials of the second kind [45,52]. In general case, for topological SSH chains analytical solutions are also possible but they have no simple transparent forms. Thus, we obtains here the local DOS along the chain for a given leads DOS numerically (using Java and Fortran codes) and compare the results for different system geometries.

In the calculations homogenous on-site energies for all chain sites are considered, $\varepsilon_{i}=\varepsilon_{0}$ and there is used the zero temperature limit. The energies are expressed in units of Γ=1 where Γ is defined as usually for a flat, $\vec{k}$-independent lead DOS of the width *w* as follows: $\Gamma =2\pi |V_{\vec{k}}{|}^{2}/w$ (which is related to the wide band limit approximation). The energy reference point is the chemical potentials of the leads, $E_{F}=0$. For experimentally available values of Γ≃0.5 eV the typical coupling units between atomic sites would be 0.5–2 eV.

## 3. Results and Discussions

### 3.1. Straight SSH Atomic Chain

**Figure 2:**
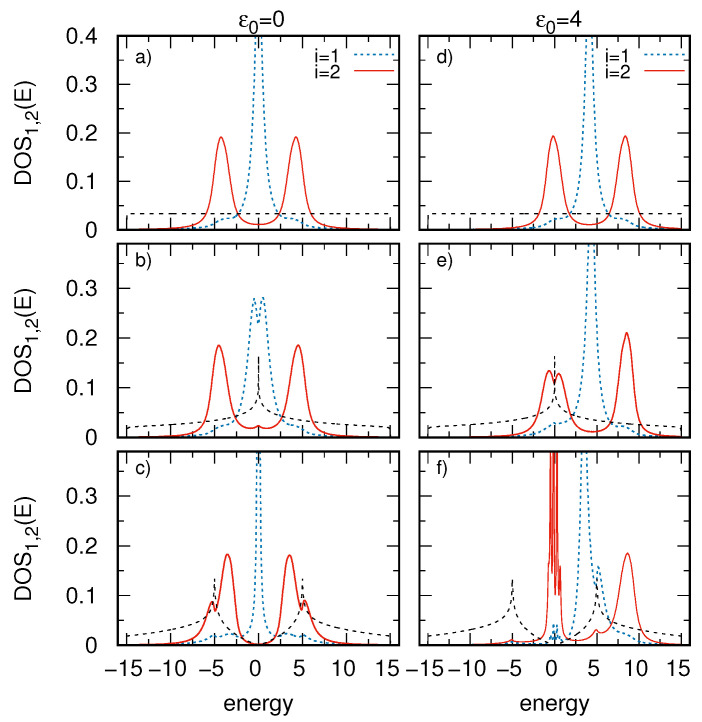
Local DOS at two sites of the straight SSH chain (see panel a in Figure 1), i=1 (blue broken curves) and i=2 (red solid curves) as a function of energy, and for different surface DOS shown by the dashed black curves: rectangular DOS (panels **a** and **d**), 2D-DOS with one van Hove singularity (panels **b** and **e**) and for 2D-DOS with two van Hove peaks (panels **c** and **f**). The left (right) panels correspond to $\varepsilon_{0}=0$ ($\varepsilon_{0}=4$). The chain length is N=12 sites, the couplings are V=1, W=4, and the surface DOS width is w=30 (all energies are expressed in Γ units).

The first system under consideration is a straight SSH chain on 2D electrode (see Figure 1, panel a). In Figure 2 we analyze the local DOS of the chain for different surfaces. In particular it is assumed a flat rectangular surface DOS (panels a and d), surface DOS with one van Hove singularity in the middle of the band (panels b and e), and the surface DOS with two van Hove singularities with vanishing value in the middle of the band (panels c and f). These functions are shown in Figure 2 (dashed black curves) and they correspond to the wide-band approximation, 2D rectangular lattice, and 2D honeycomb lattice, respectively. The surface DOS can be obtained exactly for different regular atomic latices [53,54,55,56] and their analytical forms are shown in the Appendix A. Note that such atomic lattices are fabricated in many experiments e.g., Pb atomic films form a hexagonal close-packed structure on Al(111) substrate, Sn atoms on Al(100) or Al(111) are found to form square-like structures [57,58] similarly, Sb films form square-like structures on Rh(111) [59]. In addition, on vicinal surfaces one can obtain multiple Pb chains which form regular atomic nanoribbons on each terrace of Si(553)-Au substrate [43]. We are interested in the local DOS of the chain at the edge site (where the mid-gap topological state appears, i=1, dashed blue curves) and also at one inner site with no topological states (here i=2, red solid curves). Additionally, both symmetrical and asymmetrical cases, i.e., $\varepsilon_{0}=E_{F}=0$ (left panels) and $\varepsilon_{0}=4$ (right panels) are considered.

For the SSH_1_ chain on the surface with rectangular DOS (panels a and d) the electronic structure of the chain is characterized by two sideband peaks (bulk bands) with the energy gap between them for the interior sites (e.g., for i=2) and the mid-gap state localized at $E=\varepsilon_{0}$ for the edge site (i=1 and i=N). In this case, the surface DOS is flat (energy independent) and can be effectively described within the wide-band limit approximation. Thus, the surface described within this approximation does not influence the chain topological states which is in agreement with the literature results [60]. The situation changes for realistic 2D square lattice substrate with the van Hove singularity in the middle of the band (panels b and e). This van Hove peak can be considered as a kind of sharp atomic state which is coupled with the mid-gap edge state of the SSH chain. It is the reason that the topological SSH state splits for E=0 (panel b) and it is not robust against the surface states. In addition, for the asymmetrical case (panel e, $\varepsilon_{0}=4$) the energy of the van Hove peak corresponds to the energy of the lower (left) sideband in the chain and there is a small local minimum in the structure of this band at E=0. For other energies, i.e., beyond the van Hove singularity the substrate spectral density is relatively flat and thus the chain DOS is almost the same as for the rectangular case (see the upper panels). The most interesting case one can observe for the honeycomb lattice underneath the chain (panels c and f). Now, the substrate DOS is characterized by two van Hove peaks at E=±5 and a local minimum in the middle of the band. As before these peaks are responsible for the local minima in the chain DOS, i.e., in both sidebands (panel c) or in the mid-gap state (panel f). However, for very low values of the substrate DOS (near E=0) the chain-surface effective coupling Γ(E) tends to zero which leads to the atomic limit for this energy span. Thus, for the symmetrical case the mid-gap topological state is very narrow (and almost disperssionless) than for non-zero surface DOS (shown in panels a and b). One can see that 2D substrates essentially modify topological states and change effectively their shape. For the asymmetrical case (panel f) low values of the substrate DOS correspond to the left sideband of the chain DOS. The sidebands consist of many bulk states (due to the surface coupling they form relatively smooth function of the energy) but in this case the lower one reveals an atomic structure around the zero energy and has many sharp peaks. At the same time the second (upper) sideband of the chain DOS (around E=9) remains in the form of the bulk shape. As a consequence, very regular structure of the chain DOS can drastically change in the presence of the van Hove singularity or energy dips in the substrate DOS. Note that for $\varepsilon_{0}=0$ (left panels) all local DOS functions are symmetrical in the energy scale which results from symmetrical structures of the surface DOS with respect to the Fermi energy and the particle-hole symmetry is not broken in this case. For different value of $\varepsilon_{0}$ this symmetry still exists for a plane surface DOS (right upper panel) but it is broken for real 2D lattice DOS (panels e and f). Thus, strong asymmetry in the local DOS of atomic system can come from the peaked structure of the surface DOS which was not reported before and it leads to breaking of the system particle-hole symmetry.

### 3.2. Boundary Effects in Chain DOS

**Figure 3:**
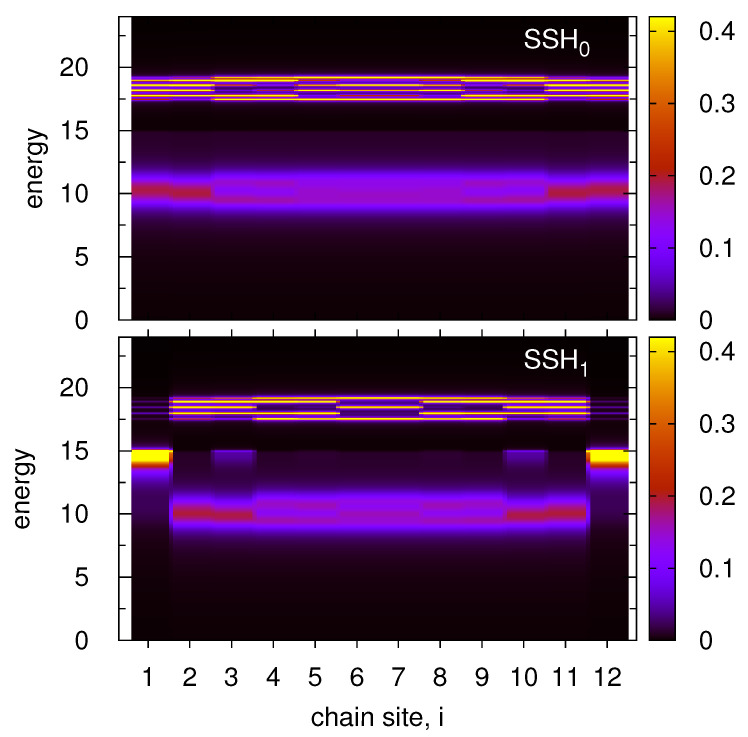
Heatmap plots of the energy and site dependent local DOS along the whole chain, N=12, for the same system as in Figure 2 for the rectangular surface DOS and for $\varepsilon_{0}=14$ (in the vicinity of the edge surface DOS). The upper (bottom) panel corresponds to the SSH_0_ chain with V=4, W=1 (SSH_1_ chain with V=1, W=4).

It was shown that local dips in the structure of the substrate DOS strongly modify electronic properties of the SSH chain for symmetrical as well as asymmetrical cases (i.e., for different positions of the chain on-site energies, $\varepsilon_{0}$). However, for larger $\varepsilon_{0}$ or for relatively narrow surface band the chain on-site energies can lie in the vicinity of the substrate DOS boundaries or even beyond the band. In such a case one expects localized states in the system (like for an adatom on the surface [50]) and significant changes in the chain DOS structure can appear. Thus, it is desirable to study this effect for topologically trivial and nontrivial SSH chains on a surface. The corresponding results are depicted in Figure 3 for both chains, SSH_0_ (upper panel) and SSH_1_ (bottom panel) in the form of heatmaps of the local DOS along the whole chain, i.e., at each chain site, i=1,...,12. The position of $\varepsilon_{0}$ is very close to the surface DOS boundary, $\varepsilon_{0}=14$, such that the bottom sideband of the SSH chain lies inside the surface DOS and the second one is outside this band. For the trivial SSH chain (upper panel) the boundary of the surface DOS (E=15) corresponds to the chain energy gap and there are still two sidebands of the chain DOS (inside and outside the surface band). The lower sideband is quite smooth (like the sidebands in Figure 2, upper panels) as its energy lies inside the surface band. On the other hand, the outside sideband is characterized by very sharp and high DOS peaks as in this case there are no corresponding energy states in the substrate and the effective coupling Γ(E)=0 for E>15. Thus, outside the substrate band the chain states tend to atomic limit and strong asymmetry of the local DOS appears (as a function of the energy). For nontrivial SSH_1_ chain (bottom panel) both chain sidebands are also nonsymmetrical and they behave in the same way as for the SSH_0_ chain. However, at both edge sites, i=1 and i=N, there exist topological states and as one can see for E>15 (outside the substrate band) these states suddenly disappear and no extra states are observed above this energy. In this case, the structure of topological state becomes asymmetrical and this interesting effect is going to be studied in more details.

**Figure 4:**
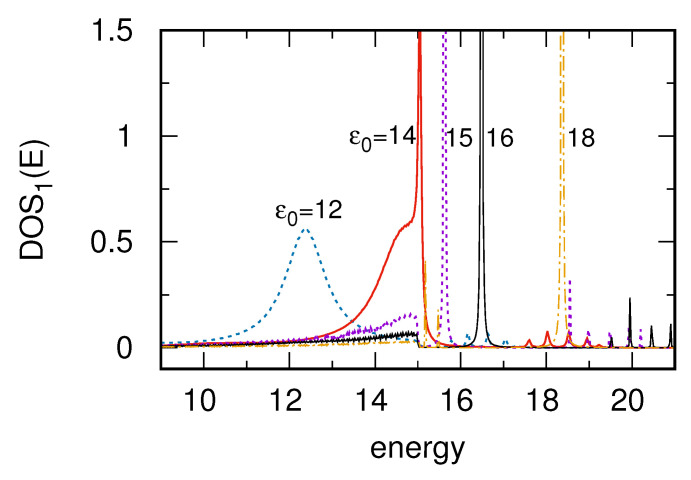
Local DOS at the first site of the SSH_1_ chain on a surface described by a rectangular DOS (the same as in Figure 2, upper panels) for different values of the chain on-site energy, $\varepsilon_{0}=12,14,15,16$ and 18, respectively. The SSH_1_ couplings are V=1, W=4.

**Figure A1:**
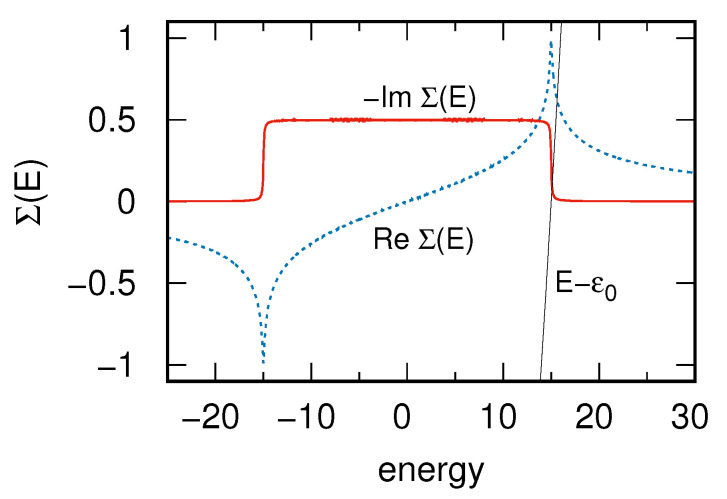
Real and imaginary parts of the Σ(E) function, ReΣ(E)=Λ(E) and ImΣ(E)=−Γ(E)/2, respectively, for a rectangular lead DOS of the width w=30. The cross point of $E-\varepsilon_{0}$ and Λ(E) indicates a localized state in the system, here it is set $\varepsilon_{0}=15$.

In order to analyze the SSH mid-gap states near the surface DOS boundary there were performed additional studies which are described in Figure 4. Here it is considered the local DOS at i=1 for different on-site energies in the chain, around $\varepsilon_{0}≃15$, as it is depicted in the legend. Note that the surface is described by a rectangular DOS with nozero constant value for |E|≤15, thus the chain on-site energies correspond to the surface band edge. For $\varepsilon_{0}=12$ the whole mid-gap state lies inside the surface DOS region and its shape is almost the same as for smaller $\varepsilon_{0}$ (see Figure 2, upper panels) or as for the surface DOS obtained within the wide band limit. Here, the maximum of this state is somewhat shifted from $\varepsilon_{0}=12$ towards higher energies due to nonzero Λ(E) function which effectively shifts the on-site energies (see Appendix B). Note that for small value of $\varepsilon_{0}$ (in the middle of the band) the local DOS peak at the edge site appears for $E≃\varepsilon_{0}$ because Λ(E) tends to zero for E=0 as was shown in Figure A1 in the Appendix B. However, for larger value of $\varepsilon_{0}$ this function increases thus the local DOS maximum is slightly shifted and appears for E=12.4 in this case ($\varepsilon_{0}=12$). For $\varepsilon_{0}=14$ the state lies closer to the band edge (red curve) and the structure of topological state drastically changes. As before there is no maximum of this state at $E=\varepsilon_{0}=14$ but it is shifted towards higher energies. However, outside the surface band only dispersionless localized states can be observed (a very high and narrow peak appears for the energy near the surface DOS edge). Its position is determined by the cross of $E-\varepsilon_{0}$ linear function with Λ(E), as was shown in the Appendix B. In consequence the mid-gap topological state assumes asymmetrical structure which consists of a wide part together with strongly localized peak (red curve). Such a shape of the edge topological state in 1D chain has not been reported before and it cannot appear in a system described by the wide band approximation. For further values of $\varepsilon_{0}$, beyond the surface band, one can observe only sharp dispersionless states of the chain local DOS ($\varepsilon_{0}=16,18$) but also some small DOS spikes are visible for E>15. These small peaks are related to the sideband structure of the local DOS in the chain. It is worth mentioning that for the SSH_0_ chain the local DOS at the edge sites has only two sidebands structure and for the SSH_1_ chain this structure still exists together with the mid-gap states.

### 3.3. Zig-Zag and Armchair-Edge Chains between 2D Systems

Thus far, there was investigated a straight SSH chain on different 2D substrates. Now the objects of study will be more complex hybrid systems composed of a chain between two 2D electrodes which are schematically shown in Figure 1 (panels b and c). We consider a simple zig-zag geometry of the SSH chain, as well as an armchair edge chain structure which can be fabricated on various vicinal surfaces. For the same lattice structure of both (left and right) leads the results are similar to those discussed in the previous sections, thus in this part there is considered two different surface leads composed of a square or hexagonal 2D atomic lattices which are characterized by the van Hove singularities.

**Figure 5:**
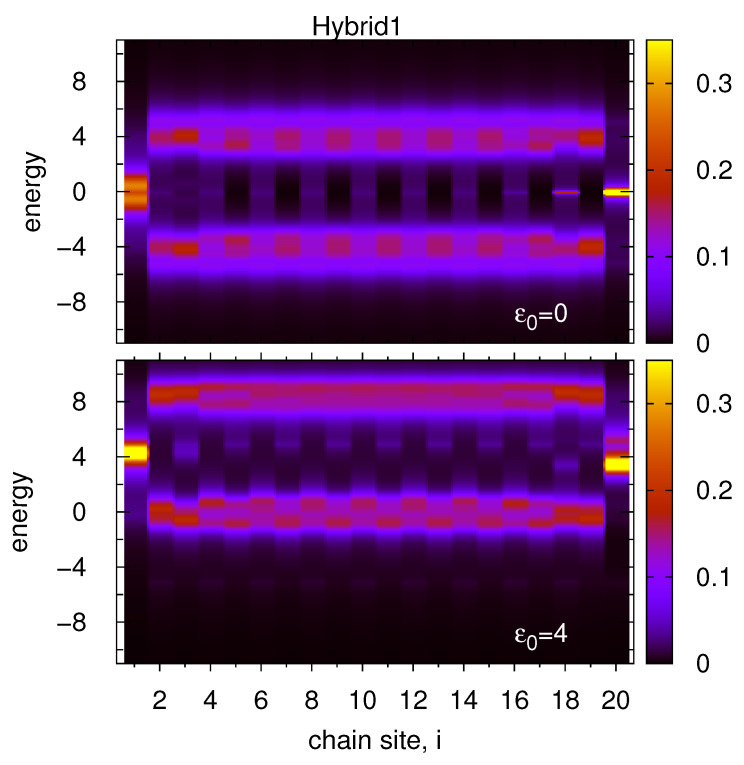
Heatmap plots of the energy and site dependent local DOS along the whole zig-zag chain for the Hybrid1 system (see panel b in Figure 1) for the on-site energies $\varepsilon_{0}=0$ (upper panel) and for $\varepsilon_{0}=4$ (bottom panel). The left (right) 2D electrode is described by DOS with one van Hove (two van Hove) singularity and the chain length is N=20 sites, V=1, W=4.

Let us first assume a simple zig-zag SSH chain which sites are coupled alternately with the left or right electrodes (so called Hybrid1 system, Figure 1, panel b). It is analysed the energy dependent local DOS at each chain site along the whole chain (N=20) for the symmetrical case (Figure 5, upper panel) and for $\varepsilon_{0}=4$ (Figure 5, bottom panel). In this system every odd chain site is coupled with an electrode characterized by a single van Hove peak in the middle of the band. Thus, for the first atomic chain the nontrivial state is relatively wide with small local minimum appearing for E=0—the same effect was discussed in Figure 2 panel b. On the other hand, every even number of atom in the chain is coupled with a honeycomb lattice electrode characterized by two van Hove peaks and minimum value of DOS in the middle of the band, thus the edge topological state at the last site, i=20, becomes narrower and higher (as was discussed in Figure 2, panel c). However, this state has some effective dispersion as it is coupled via the last but one site (and also other sites) with the left electrode for which there is a finite value of DOS at the Fermi level. It is also interesting that every second site in the SSH_1_ chain is characterized by small but nonzero DOS in the energy gap region. This effect is known in the SSH chains where topological states appear mainly at both chain ends but they also symmetrically leak inside the chain with decreasing intensities [29,46]. Here one can observe asymmetrical values of DOS along the chain. It is a consequence of much higher topological state at the last site i=20 in comparison with the first site DOS, which allows for deeper penetration of this state into the chain. As an unexpected result it was found that nonzero local DOS at the Fermi level in the chain is observed for these sites which are directly coupled with the right electrode (which has no states at the Fermi energy).

For the non-symmetrical case ($\varepsilon_{0}=4$, bottom panel) both topological states in the chain are coupled with nonzero DOS of the leads and their energy dispersions are similar to each other. However, the local DOS along the chain is not symmetrical and the maxima of both topological states appear for slightly different energies (the left one for E≃4.2 and the right one for E≃3.5). It results from the fact that the last chain site (and also every even site in the chain) is coupled with the lead characterized by two van Hove singularities (they appear for E=±5). Thus, the edge state for i=20 is split (cf. also Figure 2, panel f), and the topological peak becomes asymmetrical with shifted maximum. This effect shows that maxima of topological edge states in the SSH chain can appear at both edges for slightly different energies in 2D hybrid systems which is a new feature of topological states. It is interesting that the edge state at the first site is symmetrical (as it is coupled to the plain lead DOS around E=4) and it much more effectively spreads over the chain in comparison with the last site topological state which is strongly suppressed inside the chain due to the splitting effect. Moreover, the upper and bottom sideband structures of the local DOS are non-symmetrical in this case-the upper one is relatively smooth but the bottom one reveals periodical oscillations from site to site along the chain depending on the lead band structures. In general, the bottom sideband maxima for this system should appear near E=0 but for this energy the left lead possesses a single van Hove peak and the right lead is characterized by a local dip in DOS. Taking into account that the sites are coupled directly with only one electrode and non-directly with the second one (via the neighbouring sites), one observes mixed effects from both leads-split states with shifted maxima at every second chain site.

**Figure 6:**
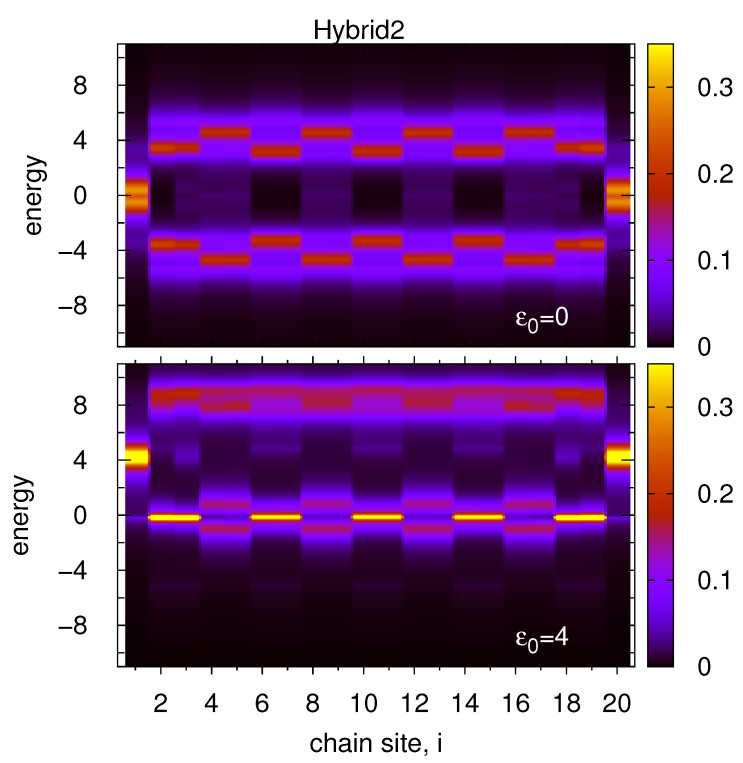
The same as in Figure 5 but for the armchair edge SSH_1_ chain (Hybrid2 geometry-see panel c in Figure 1). All parameters are the same as in Figure 5.

In the last study of this paper there is considered armchair edge geometry of the SSH chain (Hybrid2 system), i.e., every atomic dimer of the chain (two sites) is coupled alternatively with the left or right electrodes (see Figure 1, panel c). As before it is plotted energy and site dependent local DOS for two positions of the chain on-site energies $\varepsilon_{0}=0$ and $\varepsilon_{0}=4$, Figure 6, upper and bottom panels, respectively. Note that in our case the chain length is N=20, thus the first and last sites are coupled to the same left electrode which leads to fully symmetrical structure of the local DOS along the chain (they are the same for *i*-th and (N−i+1)-th sites). This armchair edge geometry is also reflected in the results depicted in both panels in Figure 6. In the upper panel the edge topological states are split and have local minimum at the Fermi energy due to the coupling with the van Hove singularity of the left lead. Two next sites are coupled to the right lead with the singularities localized at E=±5, and for these energies one observes local minima in the upper and bottom sidebands. It is the reason that the sideband maxima appear below |E|=4. The further two sites are coupled again to the left lead with relatively flat DOS around the chain sideband energy so the maxima of these sidebands are for E=±5. Note that beside the space symmetry of the local DOS along the chain there is also full energy symmetry of DOS at each atomic site with respect to the Fermi energy.

This symmetry is broken for nonzero value of the on-site energies, $\varepsilon_{0}=4$ (bottom panel). Now, the edge topological states at i=1 and i=N are described by almost symmetrical local DOS with respect to $E=\varepsilon_{0}$ but at the rest chain sites this function is non-symmetrical. The upper sideband energy lies beyond the singularities of the lead DOS and for this energy the sideband structure is relatively simple with a local maximum at E=8 (see also the right panels in Figure 2). On the other hand, the bottom sideband corresponds to the Fermi energy of the system where there are extrema of the lead band. In particular, the right lead for the zero energy has no states (local minimum in DOS); thus, the corresponding chain states tend to the atomic limit which is well visible for every two sites of the chain coupled with this electrode (light narrow peaks for E=0). The other chain sites are coupled with the left lead characterized by a single van Hove peak at the Fermi level. In this case, the bottom sideband is split leading to a local minimum in the chain DOS for this energy. In consequence, the local DOS function is asymmetrical with respect to the energy and it reveals double-site periodical oscillations in space (along the chain).

To conclude, for non-linear chain in 2D hybrid configurations one observes interesting periodical in space structures of the local DOS which reflect topological nature of the chain and singularities of both leads. In consequence, one can obtain modified topological chain with asymmetrical sideband structures and split edge states. Such a behavior makes these structures very attractive to potential applications in nanoelectronics. In the next step, it could be interesting to consider superconducting electrodes which can lead to the Majorana states in the chain and analyze time dynamics and proximity effect with topological states in 2D hybrid structures.

## 4. Conclusions

In this paper, the electronic properties of different hybrid systems composed of the SSH topological chain coupled with 2D electrodes were investigated. In particular, the mid-gap state modifications due to real structures of the surface characterized by the van Hove singularities were analysed. For rectangular or hexagonal 2D atomic lattices, such singularities in DOS often appear (like in graphene or silicene). The calculations were performed within the tight binding Hamiltonian and the Green’s function technique. The main conclusions of this work are as follows:

1. Surface with singularities in DOS essentially influences the spectral density function (local DOS) along the chain and is responsible for strong asymmetry in the topological chain energetic structure. It leads to the particle-hole symmetry breaking in the system.
2. The surface van Hove singularities can split the SSH topological state of the chain. On the other hand dips in the surface DOS lead to dispersionless strongly localized states (topological or normal) in the chain.
3. There was also discovered that topological mid-gap states can exist outside the surface band boundaries. It is important that when the chain on-site energies lie near the surface DOS edges topological state reveals partially localized behaviour with both wide dispersion due to continuous band states and sharp localized peak which comes from the surface band boundaries.
4. Different geometries of the SSH atomic chain between two 2D electrodes systems show spatial and energetic asymmetry in the structure of chain DOS which leads to different energies of both topological edge states at the chain ends.

The above findings can be verified experimentally for self-assembling or  STM-manipulated atomic chains in the SSH geometries fabricated on different 2D materials (in particular on vicinal substrates).

## Data Availability

Not applicable.

## Appendix A

The energy-band dispersion, $DOS_{s}\left(E\right)$, for the surface described by a regular tight-binding Hamiltonian can be expressed analytically by the relation:

(A1) $$\begin{array}{c} DOS_{s}\left(E\right)=\sum_{n}\int \frac{d\vec{k}}{{\left(2\pi \right)}^{2}}\delta \left[E-\varepsilon_{n}\left(\vec{k}\right)\right]\;, \end{array}$$

where the summation over *n* concerns the energy bands in the Brillouin zone with the energy dispersion, $\varepsilon_{n}\left(\vec{k}\right)$. In 2D tight-binding models e.g., for square, triangle, honeycomb, graphene-like, Lieb, Kagome and other lattices the energy dispersion is derived analytically and can be expressed by means of the elliptic integrals [53,54,55,56]. In particular for 2D rectangular lattice one has:

(A2) $$\begin{array}{c} DOS_{s}\left(E\right)=\frac{4}{\pi^{2}w}K\left(\sqrt{1-{\left(\frac{2E}{w}\right)}^{2}}\right)\Theta \left(\frac{w^{2}}{4}-E\right), \end{array}$$

where $K\left(x\right)=\int_{0}^{\pi /2}{\left(1-x\sin^{2}\varphi \right)}^{-1/2}d\varphi$, and *w* is the bandwidth of DOS. This relation is characterized by a van Hove logarithmic singularity in the middle of the band as it is shown in Figure 2 (dashed black curve, panel b). For the honeycomb lattice the structure of DOS is described by the following formulae:

(A3) $$DOS_{s}\left(E\right)=\frac{2|E|}{\pi^{2}t^{2}}\left\{\begin{array}{c} \frac{1}{\sqrt{f(|E|/t)}}K\left(\frac{4|E|/t}{f(|E|/t)}\right),\;for\;0<\left|E\right|<t\;\\ \frac{1}{\sqrt{4|E|/t}}K\left(\frac{f(|E|/t)}{4|E|/t}\right),\;for\;t<\left|E\right|<3t \end{array}\right.$$

where $f\left(x\right)={\left(1+x\right)}^{2}-{\left(1-x^{2}\right)}^{2}/4$, and t=w/6. In this case, the structure of DOS has two van Hove singularities localized at E=±t with a local minimum in the middle of the band, which is shown in Figure 2 (dashed black curve, panel c). It is worth noting that in three dimensions a simple cubic lattice is characterized by a flat dispersion of DOS in the middle of the band thus a rectangular DOS (or even wide band limit approximation) relatively well describes this structure.

## Appendix B

Localized states which can appear at the chain sites are related with the Green’s function determinant in the form $E-\varepsilon_{0}-\Sigma \left(E\right)$, where Σ(E) is expressed by the relation below Equation (5) and satisfies the relation: Σ(E)=Λ(E)−iΓ(E)/2. These functions, Λ(E), Γ(E), are related through the Hilbert transform:

(A4) $$\begin{array}{c} \Lambda \left(E\right)=\frac{1}{2\pi }\int_{-\infty }^{\infty }\frac{\Gamma (E^{\prime })}{E-E^{\prime }}dE^{\prime }, \end{array}$$

thus the knowledge of Γ(E) which depends in the substrate DOS structure allows one to determine all other functions. Moreover, it is also possible to predict the appearance of localized states in the system. These states are observed for vanishing Γ(E) (outside the surface band) and they appear for minimal value of $E-\varepsilon_{0}-\Lambda \left(E\right)$, i.e., for $E-\varepsilon_{0}=\Lambda \left(E\right)$. In this case, Λ(E) function renormalizes the on-site energy position leading to the localized state outside the substrate DOS. It can be shown graphically in Figure A1, where Λ(E), Γ(E) and $E-\varepsilon_{0}$ functions for a rectangular lead DOS are analysed.

As one can see Γ(E) function (red solid curve) is strictly related to the lead DOS. Its Hilbert transform, ImΣ(E)=Λ(E) (broken blue curve), is nonzero even for vanishing Γ(E), and the localized state appears for the crossing point of $E-\varepsilon_{0}$ and Λ(E) functions.
